# Impacts of ocean acidification on intertidal benthic foraminiferal growth and calcification

**DOI:** 10.1371/journal.pone.0220046

**Published:** 2019-08-21

**Authors:** Fabricio Guamán-Guevara, Heather Austin, Natalie Hicks, Richard Streeter, William E. N. Austin

**Affiliations:** 1 School of Geography and Sustainable Development, University of St. Andrews, St Andrews, Scotland; 2 School of Biological Sciences, University of Essex, Colchester, United Kingdom; 3 The Scottish Association for Marine Science (SAMS), Oban, Scotland; Victoria University of Wellington, NEW ZEALAND

## Abstract

Foraminifera are expected to be particularly susceptible to future changes in ocean carbonate chemistry as a function of increased atmospheric CO_2_. Studies in an experimental recirculating seawater system were performed with a dominant benthic foraminiferal species collected from intertidal mudflats. We investigated the experimental impacts of ocean acidification on survival, growth/calcification, morphology and the biometric features of a calcareous species *Elphidium williamsoni*. Foraminifera were exposed for 6 weeks to four different pH treatments that replicated future scenarios of a high CO_2_ atmosphere resulting in lower seawater pH. Results revealed that declining seawater pH caused a decline in foraminiferal survival rate and growth/calcification (mainly through test weight reduction). Scanning electron microscopy image analysis of live specimens at the end of the experimental period show changes in foraminiferal morphology with clear signs of corrosion and cracking on the test surface, septal bridges, sutures and feeding structures of specimens exposed to the lowest pH conditions. These findings suggest that the morphological changes observed in shell feeding structures may serve to alter: (1) foraminiferal feeding efficiency and their long-term ecological competitiveness, (2) the energy transferred within the benthic food web with a subsequent shift in benthic community structures and (3) carbon cycling and total CaCO_3_ production, both highly significant processes in coastal waters. These experimental results open-up the possibility of modelling future impacts of ocean acidification on both calcification and dissolution in benthic foraminifera within mid-latitude intertidal environments, with potential implications for understanding the changing marine carbon cycle.

## Introduction

The partial absorption of atmospheric CO_2_ by the ocean (up to 40% of total CO_2_ emissions) since industrial times has progressively changed seawater chemistry through a process known as ocean acidification (OA) [1–5]. As a result, seawater pH, carbonate ion concentration [CO_3_^2-^], and saturation state (Ω) with respect to carbonate minerals have been declining and are now known to affect mainly calcifying organisms across different trophic levels [1,6–12]. Changes in growth, calcification, metabolism and survival by marine calcifiers appear to be the most significant biological responses to OA [13–17]. In some cases, however, the impact of OA on marine biodiversity is markedly different due to the natural variability among individuals, species, communities, and ecosystems [4,5,18–20]. Besides these general differences observed at different functional levels, there is growing concern regarding potential shifts in the size and species composition of multiple marine communities. This ecological succession via loss of marine biodiversity, where only a few species become beneficiaries of changes in seawater chemistry, may occur under future increased CO_2_ concentrations [3]. The magnitudes of these shifts in the community structure remain unclear due to the complexity of marine biological systems mainly controlled by multiple abiotic and biotic drivers [3,21,22].

These potential modifications in community composition and energy flow, via a shift in trophic dynamics, may alter carbon cycling and ecosystem productivity of different environments [3,21,23]. For instance, OA may cause a shift in foraminiferal benthic community structure, given the likelihood of greater ecological advantage to non-calcifiers over calcifying species in coastal benthic habitats in the long-term [23]. This shift in species assemblages has been observed in natural shallow-water CO_2_ seeps where a gradient in calcium carbonate saturation exists and where assemblages of foraminifera species shifted from calcareous species to agglutinated species as pH seawater levels naturally reduced [24]. There is still a lack of understanding of the ecological mechanisms which generate early foraminiferal succession processes, such as the time required for benthic organisms to display significant changes in multiple biological parameters as a response to changing pH, and the optimal target species for monitoring these changes remain elusive.

Benthic foraminifera, a group of protozoa with calcareous, siliceous, agglutinating or organic walled tests [25] are capable of inhabiting diverse marine environments due to their broad ecological adaptability to environmental changes, which in turn control their distribution and abundance [26–28]. However, their presence and biological role in marine sediments may be severely affected by elevated CO_2_ and a reduction in the availability of carbonate ion [CO_3_^-2^] [29]. These marine organisms play a key role in biogeochemical cycles due to their ability to degrade large amounts of organic matter available in shallow-water sediments [6]. Furthermore, their importance in carbon cycling, especially through calcification, is highly significant in coastal waters where they may contribute up to 30% of total CaCO_3_ production [30,31].

Multiple studies have assessed the effects of changes in seawater chemistry on benthic foraminifera, demonstrating that pH changes can strongly influence biometric and morphological features of foraminiferal test (e.g. size/diameter, weight, functional feeding structures, etc.) with an ultimate effect on the growth and calcification rates and biomass of benthic foraminifera, especially in shallow water areas [4,20,23,31–36]. Much of this research has focused on the use of benthic foraminifera from coral reef habitats [32,35–40]. Whilst this research improves the ability to accurately predict ecological responses under elevated CO_2_ concentrations [41], it is limited to coral reef ecosystems and not representative of many other coastal sediment habitats.

In contrast, little information is available for important coastal environments such as tidal flats; such non-charismatic coastal ecosystems are widely recognized as requiring greater research effort [42,43]. Here, resident benthic communities may exhibit different vulnerability levels to future changes in the ocean carbonate chemistry as a function of changes in atmospheric CO_2_.

Research that assesses multiple biological parameters of dominant species may provide evidence on which co-existing benthic foraminiferal species are likely to be more vulnerable to short or prolonged periods of high CO_2_ concentrations. Furthermore, this may enhance the understanding of how individual and ecosystem-scale responses will occur under future high CO_2_ concentrations. These biological responses, in some cases, might be markedly different from globally predicted scenarios for year 2100. In this study, however, we investigate whether the dominant, low-Mg calcite benthic foraminifera *Elphidium williamsoni*, typically found in temperate intertidal cohesive sediment, is negatively impacted by short-term exposure to various increased CO_2_ concentration conditions, simulating predicted future climate change scenarios. Potential alterations in growth-related biometric parameters, test morphology and calcification process as a response to OA conditions may have important ecological implications for future mid-latitude intertidal environments.

## Materials and methods

### Collection site and sampling

Sediment scrapes (~ 1cm depth) containing benthic foraminifera were collected from intertidal mudflat in the Eden Estuary, Fife, N.E. Scotland (56°22’N, 2°50 W) during low tide, in late July 2015 (S1 Fig). Gavin Johnson, Scottish Natural Heritage, provided permission for site access and sampling on the Eden Estuary Site of Special Scientific Interest. In the laboratory, all sediment samples were mixed and sieved over a set of 63 μm and 500 μm screens. The sieved sediment fraction was left to settle in plastic containers for three hours. A small subsample of this sediment was examined through a stereoscopic binocular microscope to confirm that living foraminiferal specimens of *E*. *williamsoni* were present; which comprised up to 80% of the living benthic foraminiferal assemblage. This species is easily identified through the yellow/brown-coloured protoplasm extensively distributed across the entire foraminiferal tests, except in the last chambers [25,44]. Previously, the naturally intense protoplasm colour has been widely used as an indicator of viable foraminiferal individuals [45], and additional observation of pseudopodia activity confirmed that these individuals were alive. Subsequently, approx. 100 cm^3^ of mixed sediment containing 10–20 live specimens/cm^3^ was placed in a series of 500 cm^3^ filtering flasks. The number of specimens used for this experiment was approx. 20000 live specimens. These glass containers were filled with filtered natural ~33 salinity seawater containing a final concentration of 10 mg/L of the fluorescent marker calcein [45,46]. Each flask was sealed with a rubber stopper with three inlets on top, one for air tubing and two to allow seawater with calcein to be continually recirculated into and out of the flask through a 1 L reservoir glass bottle. Multi-channel peristaltic pumps controlled the flow between the experimental flasks and the calcein bottles. The calcein-seawater solution was changed weekly. This seawater calcein incubation was left running for 5 weeks in a temperature-controlled room at a constant temperature of 13°C which was the equivalent minimum summer temperature recorded. The light condition was a 12:12-h light: dark cycle (S2 Fig). Fortnightly sampling observations using a fluorescence microscope provided information on the incorporation process of calcein into the newly calcifying foraminiferal tests.

When the calcein incubation period was concluded (5 weeks), selected live specimens were examined as described above. Surviving live specimens were picked out and cleaned of any detritus attached to their shells (tests) using a fine paintbrush, and used for subsequent CO_2_ experiments as detailed below.

### Experimental setup

Calcein-labelled specimens were randomly selected and transferred into foraminiferal culture chambers containing a tissue cell culture insert with a silica layer [29,43,44]. As specimens of *E*. *williamsoni* are frequently found within sediments with a significant clay/silt content [25], silica was used as an artificial sediment in a similar size-range to the fine sediment fraction (clay and silt) naturally found in the Eden Estuary (< 63μm).

Culture chambers were connected to a manipulative mesocosm (controlled recirculating seawater is shown in S3 Fig). There were four culturing chambers for each pH treatment, as supplied by the mixing tank, and each chamber contained ~250 foraminifera (n = 1000 per treatment). Prior to the 6-week-experimental period, a time period of 10 days for acclimation was carried out to prevent any “shock” response from the foraminiferal individuals due to a sudden change in pH. During the acclimation time, except for the mixing tank with seawater at a natural pH of 8.1, the seawater pH of the remaining mixing tanks was reduced by approximately 0.1 units per day until each target pH level was attained, ensuring that the measured responses were due to the treatments. Thus, specimens cultured at the lowest pH values required a longer time period for acclimation (e.g. but no longer than 10 days in total) compared with those cultures incubated at a pH close to natural seawater (pH 8.1).

The seawater pH was continually manipulated by bubbling air with a known equivalent atmospheric concentration of CO_2_, using BOC industrial grade CO_2_ (approx. 400, 600, 900 and >2000 μatm *p*CO_2_) into 4 mixing tanks, respectively. Thus, the four selected pH levels (total scale) of 8.1 (ambient), 7.9, 7.7 and 7.3 represent the range of pH predicted for future scenarios for the years 2100 and 2300 [47,48]. Seawater was continually pumped from the 400 L mixing tanks (following the terminology of Cornwall and Hurd, 2016 [49]) into the culturing chambers of each treatment (n = 4) through peristaltic pumps at a rate of 30 mL/min. This experimental design has been used for similar foraminiferal experiments [33,50,51]. All analyses on treatment effects combined the data from each of the ~1000 individuals by treatment, partly due to the high mortality rate, ensuring the data is then pooled by treatment and removes any effect of individual culturing chambers. The pH (total scale) and temperature of seawater in the mixing tanks were continually monitored throughout the experimental period using a pH and temperature controller (IKS Aquastar, IKS ComputerSysteme GmbH, Germany) via 4 pH modules and 4 temperature modules, one placed in each mixing tank. Additional measurements of pH, temperature, and salinity were recorded manually at fortnightly intervals via additional probes. The temperature and pH (total scale) were measured at 13°C using a Mettler Toledo Seven Multi pH meter with a pro-glass electrode. This probe was calibrated using pH buffers 4.00, 7.00 and 10.00. Measures of salinity were performed using an Orion 3-Star Plus benchtop conductivity meter kit with a standard 1413 μS and a pro-glass electrode providing a relative accuracy of 0.1 ppt (see S6 Table).

### Carbonate system parameters

Three replicate seawater samples from each tank of the mesocosm were taken throughout the experiment at fortnightly intervals to measure total alkalinity (TA). These samples were stored in borosilicate glass Labco exetainer vials (12 mL) and poisoned with 50 μL of mercuric chloride (HgCl_2_). Vials were kept under refrigeration (4 °C) prior to analysis at the Scottish Association for Marine Science (SAMS). Total Alkalinity (TA) concentrations of the seawater samples were analysed at 25°C using an automatic potentiometric 196 titrator (888 Titrando, Metrohm, Switzerland) with Tiamo V 2.1 software [52,53]. A three-point calibration was carried out using buffer solutions pH 4, 7, and 9 (Metrohm UK Ltd.) before TA analysis. The precise volume of HCl acid added during titration was plotted against pH; the resulting curve was subsequently logged to obtain a straight line. The gradient of this straight line was used to calculate TA [54]. The accuracy of the titrator was monitored by using a certified CO_2_ reference material (Andrew G. Dickson, Scripps Institution of Oceanography, CA, United States) [55]. The measured values of temperature, salinity, pH and total alkalinity (TA) were used to calculate the carbonate system parameters such as dissolved inorganic carbon (DIC), *p*CO_2_, bicarbonate ions (HCO_3_^-^), carbonate (CO_3_^-2^) concentration and saturation states of calcite (Ω_Calcite_) and aragonite (Ω_Aragonite_) using CO_2_sys.xls (version 01.05) [56].

### Foraminiferal feeding process

During the calcein incubation and throughout the entire experiment, the foraminifera were fed weekly with ~10μL/cm^2^ of each of the algae *Dunaliella tertiolecta* and *Rhodomonas salina* (typically 1×10^7^ cells ml^−1^). Concentrated algal solutions were defrosted prior to use for foraminiferal feeding. Both algal species were axenic clones provided by the Culture Collection of Algae and Protozoa (CCAP) at SAMS.

In the manipulative mesocosms, peristaltic pumps were switched off during the feeding procedure for 2 hours to allow algae to settle and also to avoid loss of this food material by resuspension when the system was restarted. The feeding procedure itself involved using a syringe to add the algae to the chambers through one of the free ports. All foraminifera in all chambers were fed at approximately the same time each week.

### Biological parameters

After completing the experimental period, all chambers were opened up and inserts were removed and placed onto 6-well plates. Subsequently, foraminiferal individuals were picked out and transferred into clean petri dishes and washed carefully with distilled water to remove any excess silica and food cells. All specimens of *E*. *williamsoni* were individually mounted on 32-hole micro-palaeontological cardboard slides; individual foraminifera were assigned a unique identification number.

Relative abundance distributions of live and dead foraminiferal individuals were determined according to whether or not new chambers (post-calcein incubation) were added. Newly deposited chambers, maximum diameter and weight of live specimens were used to estimate the survival rate, growth and calcification across the different pH conditions.

### Maximum test diameter and test weight

Measurements of maximum test diameter (μm) and dry test weight (μg) of each individual specimen (n = 3528) were recorded at the end of the experiment. A microbalance (Sartorius M2P Microbalance, with a precision of ±1μg) was employed to weigh foraminiferal tests. The microbalance was tested prior to use over several days in a controlled trial to reduce the error associated with any changes either in temperature, pressure or air flow in the air-conditioned weighing room. Subsequently, using a pre-weighed aluminium capsule, each foraminiferal specimen was individually weighed three times on three different days and its overall average was used for further analysis. Average standard deviations calculated for the three dry weight measurements of foraminiferal tests in each pH treatments are pH 8.1 (+/-0.5 μg); pH 7.9 (+/-0.7 μg), pH 7.7 (+/-0.4 μg) and pH 7.3 (+/-0.9 μg).

### The shell size-normalized weight (SNW)

The shell size-normalized weight (SNW) was calculated by dividing recorded measurements of dry test weight (μg) of each foraminiferal specimen by its maximum test diameter (μm), as below. The SNW was calculated across the different culture conditions as a good indicator of test thickness or density because it removes the influence of foraminiferal test size on weight [4,57,58]; however, we note that a recent paper [59] suggests a strong effect of test size on SNW.

$${\text{SNW}}_{\text{specimen}}=\frac{\text{dry}\;\text{test}\;\text{weight}\;(\mu \text{g}{)}_{\text{specimen}}}{\;\text{maximum}\;\text{test}\;\text{diameter}\;(\mu \text{m}{)}_{\text{specimen}}}$$

### Newly deposited chambers and chamber addition rate

Observation and counting of the newly formed chambers added after the calcein incubations was carried out using a Nikon epifluorescence microscope. Chambers precipitated in the last whorl were easily recognized by their characteristic non-fluorescent colour because they grew in seawater without exposure to calcein-labelling (S4 Fig). Only individuals that showed clear evidence of one or more new chambers deposited during the experimental period (post-fluorescent growth) were considered live individuals and are referred to hereafter as live individuals. This criterion was applied to discern recently active growth within the experimental environment.

Due to the large number of specimens and the limitations of the mesocosm design that prevented repeat sampling, continuous measurements of maximum test diameter (μm) and dry test weight (μg), both normally used as indicators of changes in foraminiferal growth, were not measured through the experimental period. Instead, foraminiferal growth was inferred via estimates of chamber addition rates in each pH treatment. These measures are based on the average numbers of chambers deposited for all live individuals in each culture condition and divided by the total number of experimental days (42).

### Scanning electron microscopy (SEM) images

From each treatment, seven live specimens with intact tests were mounted onto SEM stubs using double-sided adhesive tabs after test measurement and weighing. An Emscope SC 500 sputter coater was used to coat specimens with a thin layer of gold prior to imaging with a Jeol JSM-35CF SEM [23,60].

### Statistical analysis

A nested one-way ANOVA was conducted to test pseudo-replicates tanks effects of pH treatments on multiple biological parameters (S1 Table). When the analysis did not show significant interactions, further analyses were also conducted. Non-parametric tests were conducted since the assumptions of normality (Shapiro-Wilk test) and homogeneity (Levene test) were not met (p < 0.05) (S2 Table). A Kruskal-Wallis rank sum test was performed to establish whether the maximum diameter, weight of tests, or number of deposited chambers changed in response to the different pH treatments after 42 days. A Dunn's-test for multiple comparisons of independent samples was applied following the Kruskal-Wallis test to determine any significant differences between the pH treatments. Experimental pH conditions were treated as a fixed factor. The null hypothesis assumed there was no significant difference between the ambient treatment and the other experimental treatments. The relationship between maximum diameter (size) and weight was investigated by log-transforming the data of both measured variables [35]. The resultant slopes of linearized functions of each treatment were compared using a Student’s test [35]. All statistical analyses were carried out in the statistical programme R [61], and the packages MASS [62], CAR[63] PMCMR [64] were used.

## Results

From a total of 4000 live specimens transferred into the culturing chambers, split evenly at 1000 specimens per treatment at the start of the experiment, 3528 specimens (live and dead) were retrieved at the end of the experiment. This indicated a loss of 472 individuals throughout the experimental period, being greater at pH 8.1 (ambient) and pH 7.3 (see Table 1). This can likely be attributed to the passive migration of specimens out of the culturing chambers due to sporadic changes in pressure within the recirculating system [33]. Further analysis was carried out on the remaining 3528 specimens.

**Table 1:** Number of individuals of *Elphidium williamsoni* cultured under different pH conditions (pH 8.1 (ambient), pH 7.9, pH 7.7 and pH 7.3).

	Total number of individuals					
pH conditions	Start of experiment	End of experiment		Survival rate (%)	Total Mortality rate (%)	Mortality rate by OA (%)
		Retrieved/Analyzed	Alive (post-fluorescent growth)			
8.1 (ambient)	1000	801	373	46.6	53.4	0.0
7.9	1000	952	235	24.7	75.3	21.9
7.7	1000	945	194	20.5	79.5	26.0
7.3	1000	830	111	13.4	86.6	33.2

Individuals showing post-fluorescent growth throughout the experimental period were considered as live individuals. Survival rate (%) was calculated based on the number of live and dead over the experimental period.

### Live and dead foraminiferal abundance and survival rate

**Fig 1:**
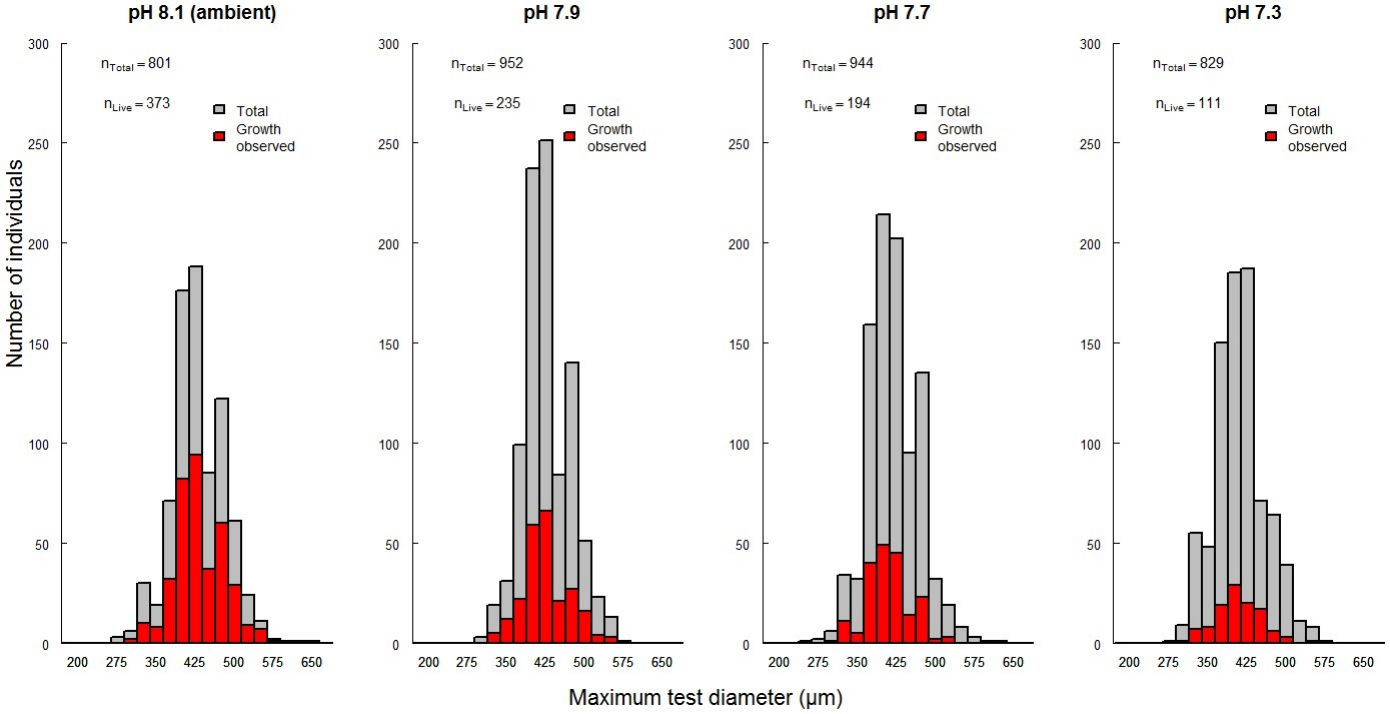
The total number of individuals of *Elphidium williamsoni* sorted into size classes after being collected at the end of the experimental period in each culture condition (pH 8.1 (ambient), pH 7.9, pH 7.7 and pH 7.3). The individuals analysed (n_Total_) and live specimens (n_Live_) observed are shown in grey and red, respectively. Bandwidth for each size class was 25 μm.

All remaining individuals of *E*. *williamsoni* were sorted into a size class of 25 μm increments according to their maximum test diameter. A size distribution chart showed the greatest abundance of both total retrieved and live individuals was found in the size class of 400–425 μm for all treatments (Fig 1). Individuals cultured at pH 8.1 displayed the largest number of surviving specimens (n_Live_ = 373) followed by treatment pH 7.9 (n_Live_ = 235) and treatment at pH 7.7 (n_Live_ = 194). In contrast, individuals cultured at pH 7.3 showed the lowest number of surviving specimens (n_Live_ = 111) (Fig 1 and Table 1).

Survival rate (SR) was calculated as a percentage of the total number of surviving individuals compared to the total number of retrieved individuals at the end of the experiment for each treatment as shown in Table 1. Specimens of *E*. *williamsoni* cultured for 6 weeks at the ambient pH of 8.1 exhibited mortality rates as high as 50% (Table 1). This mortality rate is similar to that observed throughout calcein incubation period of 4 weeks. Mortality rate (%) directly linked to an OA treatment effect was calculated by subtracting the total mortality (%) observed in each pH condition from mortality observed at ambient condition (pH 8.1). These values showed a considerable contribution of OA treatment to total mortality at low pH/ high CO_2_ concentration by up to 30% (Table 1).

Live specimens showed different levels of morphological response to pH treatment during the experimental period (S5 Fig); only specimens in good overall morphological condition (intact tests) were therefore selected for further analyses.

### Growth and calcification of live individuals

Biometric parameters of live individuals included maximum test diameter; dry test weight; and the number of new chambers added (post-fluorescent growth), were all measured after 6 weeks of culture in different pH conditions (Table 2 and S6 Fig).

**Table 2:** Maximum shell diameter (μm), shell weight (μg), and the number of chambers added and their standard deviation and standard error of the mean for *Elphidium williamsoni* across four different pH treatments.

Measured variables	pH conditions	Shell features					n
		Min.	Max.	Mean	Standard Deviation (1σ)	Standard error of mean	
Maximum test diameter (μm)	8.1 (ambient)	335.80	657.50	454.80	45.39	3.01	227
	7.9	349.80	559.60	444.20	42.41	3.43	153
	7.7	335.80	531.60	427.40	39.30	3.80	107
	7.3	363.70	559.60	439.80	45.50	8.04	32
Test weight (μg)	8.1 (ambient)	6.30	48.00	16.07	5.29	0.35	227
	7.9	7.30	32.70	15.86	4.85	0.39	153
	7.7	4.70	27.30	13.39	4.12	0.40	107
	7.3	6.30	20.30	12.21	4.09	0.72	32
Number of chambers added	8.1 (ambient)	1.00	11.00	4.04	2.28	0.15	227
	7.9	1.00	12.00	5.12	2.57	0.21	153
	7.7	1.00	9.00	3.82	2.21	0.21	107
	7.3	1.00	8.00	4.03	1.93	0.34	32

The largest maximum test diameters were found in the treatment at pH 8.1 (ambient) followed by treatment at pH 7.9, pH 7.3 and pH 7.7, respectively (Table 2). Kruskal-Wallis and Dunn's-tests revealed a statistically significant reduction by up to 17% in the mean maximum test diameter at pH 7.7 in comparison to the mean diameter observed in specimens cultured at a pH of 8.1 (p <0.001) (S3 and S4 Tables).

The heaviest test weights were found in the treatment at pH 8.1 (ambient) followed by treatment at pH 7.9, pH 7.7 and pH 7.3, respectively (Table 2). The difference in mean test weights from the pH 8.1 treatment are as follows: pH 7.9 = 1.3%; pH 7.7 = 16.6%; and pH 7.3 = 24.0%. There was a statistically significant reduction in the mean test weight across different pH conditions, especially in cultured specimens at the two lowest pH levels (*p* <0.001) (S3 and S4 Tables).

Individuals with a larger number of new chambers deposited during the experimental period were found at the lowest CO_2_ treatments (pH 7.9 and pH 8.1 (ambient)), followed by, in increasing CO_2_ level, treatment pH 7.7 and pH 7.3 (Table 2). Despite the overall trend of a decreased number of newly deposited chambers at the lowest pH conditions (Fig 1 and Table 1), this change was not significantly different to the other treatments (*p* > 0.05), except for individuals cultured at pH 7.9 (*p* < 0.001) (S3 and S4 Tables). The test weight data was subsequently used for estimation of growth rate for the duration of the experimental period.

#### Chamber addition rate

**Fig 2:**
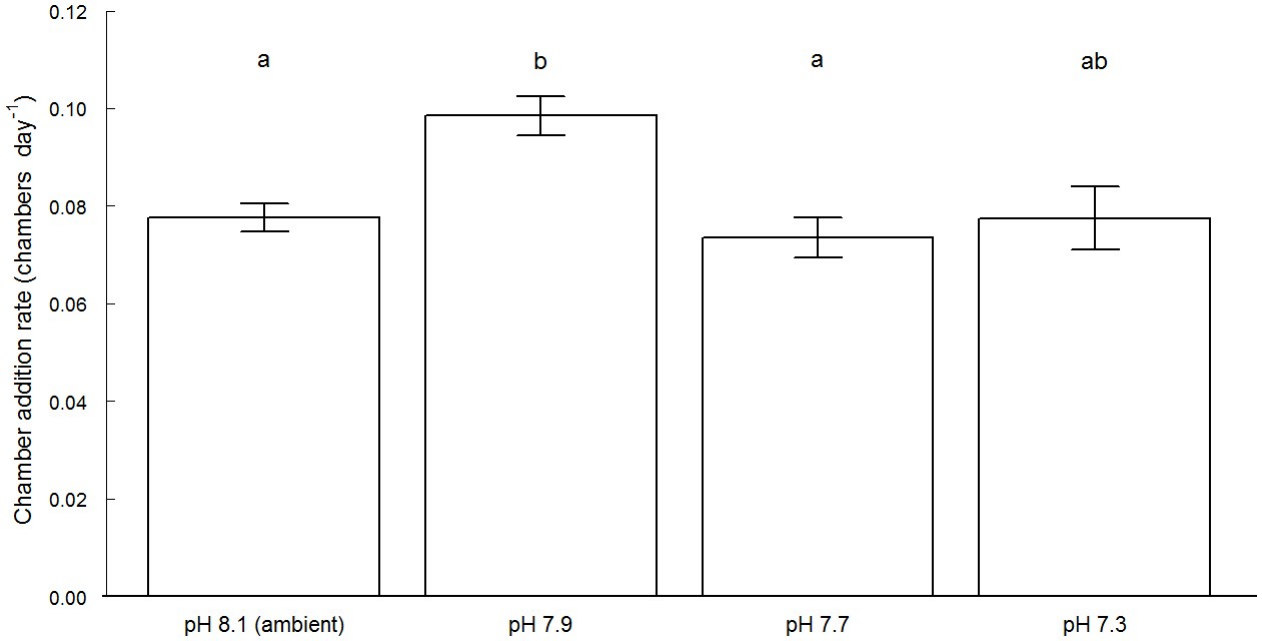
Mean values (± standard error) of the chamber addition rate for *Elphidium williamsoni* cultured at different pH conditions for an experimental period of 42 days. Treatments with significant differences are indicated by different letters (i.e. a and b) above bars at p < 0.05. Treatments with shared letters (i.e. ab) above bars indicate no significant differences (p > 0.05) observed between groups according to the Dunn's-test.

There was a slight difference in this mean chamber addition rate across the different pH treatments, but these were not statistically significant (*p* > 0.05), except for individuals cultured at pH 7.9 (*p* < 0.001) which showed a slight increase in growth rate (Fig 2).

#### Relationship between test weight and maximum test diameter

Despite the observation of a slight difference among slopes of shell weight and diameter among treatments, this difference was not statistically significant except when comparing treatment at pH 7.9 and pH 7.7 (S7 Fig and S5 Table).

#### Dry test weight vs size-normalized test weight (SNW)

**Fig 3:**
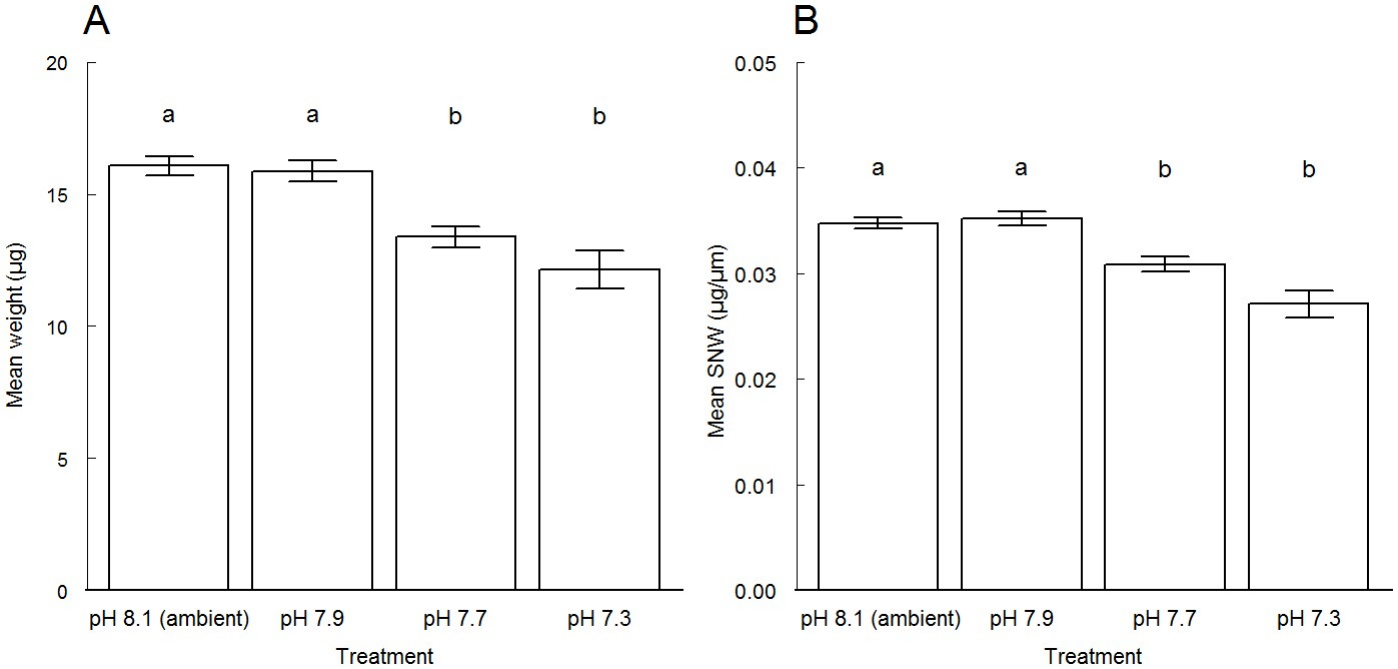
Mean values (± standard error) of (A) weight and (B) size-normalized test weight (SNW) for *Elphidium williamsoni* cultured at different pH conditions. Treatments with significant differences are indicated by different letters (i.e. a and b) above bars at p < 0.05, according to the Dunn's-test.

Dry test weight (Fig 3A) and size-normalized test weight (SNW) (Fig 3B) of *E*. *williamsoni* were both significantly reduced across the pH treatments. The lowest dry test weight and SNW were measured at the lowest pH treatments.

#### Relationship between the size-normalized weight (SNW) and carbonate ion concentration in seawater

**Fig 4:**
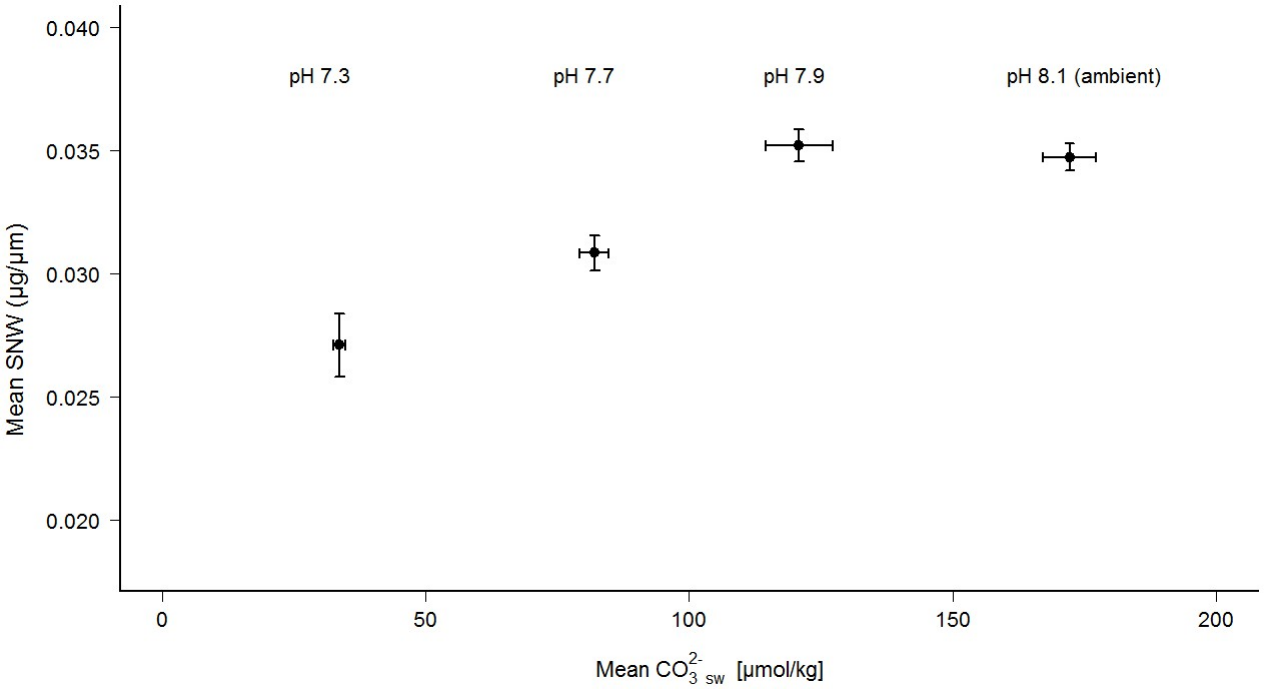
Mean values (± standard error) of carbonate ions concentration in seawater and size-normalized weight (SNW) for *Elphidium williamsoni* cultured at different pH conditions.

The relationship between the size-normalized weight (SNW) of *E*. *williamsoni* specimens and carbonate ion concentration in seawater with different pH levels is shown in Fig 4. Measurements of total alkalinity were used to calculate the mean values of carbonate ions concentrations (S6 Table). The lowest mean SNW corresponded to lowest pH conditions with the lowest mean carbonate ion concentrations in seawater. There was a positive correlation between mean size-normalized weight (SNW) and mean carbonate ion concentrations (Pearson Cor. coeff = 0.91, *p*-value = 0.08).

### SEM observations of morphological response

**Fig 5:**
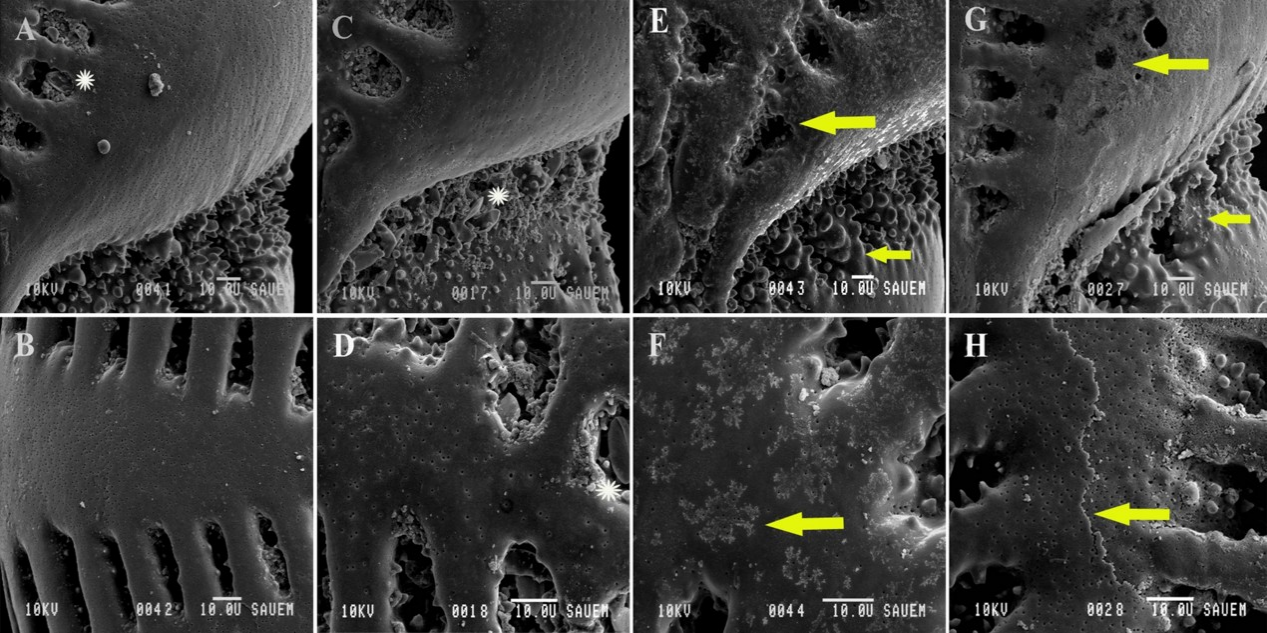
Scanning electron micrographs (SEM) images of live specimens of *Elphidium williamsoni* cultured at pH 8.1 (A & B), pH 7.9 (C & D), pH 7.7 (E & F) and pH 7.3 (G & H). (A) SEM image of side view of the apertural region showing numerous teeth and tubercles. A frustule of the diatom species *Navicula sp*. and organic material detritus are visible by a septal bridge. (B) Higher magnification of the test surface of specimen A. (C) SEM image of side view of apertural region, showing numerous teeth and tubercles with some impaled frustules of the diatom species *Navicula sp*. (D) Higher magnification of the smooth test surface of specimen C. (E) SEM image of side view of the apertural region, where signs of dissolution and cracking are clearly observed. Teeth and tubercles are less sharp with rounded shape. No frustules of diatom species are observed. (F) Higher magnification of the test surface of specimen E affected by dissolution and cracking processes. (G) SEM of side view of the apertural region showing a reduction in the number of teeth and tubercles. Dissolution and cracking processes are clearly observed in multiple structures with a severe effect on septal bridges and sutures. No frustules of diatom species are observed. (H) Higher magnification of the test surface of specimen G, showing several test wall layers, septal bridges and sutures affected by dissolution and cracking processes. (*) White asterisks show the presence of diatom *Navicula sp*. ← Yellow arrows show areas affected by dissolution.

SEM images of *E*. *williamsoni* showed morphological differences among specimens cultured at different pH conditions (Fig 5). These observations indicated a progressive alteration of the foraminiferal morphology (test) when individuals were exposed to high CO_2_ concentrations for the duration of the experiment.

The most significant features observed on the test surface are the presence of cracks and signs of dissolution on individuals exposed to the lowest pH levels. Specimens cultured at pH 7.7 and 7.3 displayed clear visual evidence of dissolution around the apertural region, particularly visible on some apertural teeth (Fig 5). In addition, the outermost chambers of foraminiferal specimens cultured at pH 7.7 and pH 7.3 displayed larger and irregular septal bridges and sutures with a clear sign of corrosion (cracking) compared to those cultured at pH 8.1 and pH 7.9, which exhibited smooth surfaces and regular shapes of these structures (Fig 5A–5D). In addition, newly formed chambers on surviving individuals cultured at these low pH levels were extremely fragile, and prone to breakage during the picking and cleaning processes prior to SEM analysis. This suggests a reduction of wall thickness in recently deposited chambers, which was confirmed by SNW estimations for each culture treatment, especially at the lowest pH conditions. These results collectively indicate a negative impact from lowered pH upon the calcification process.

## Discussion

Foraminifera live in a wide range of habitats across the world’s oceans as both pelagic and benthic organisms. Their ubiquitous distribution is attributed to their broad ecological adaptability to changing environmental stressors that control their distribution and abundance [26]. At the field sampling location, the natural pH ranged from 7.5 to 8.2 [65]; our experimental set-up was designed to reproduce the natural variability in pH, with experimental values within the range 7.3 to 8.1. In this study, however, experimental evidence revealed that declining seawater pH negatively affected foraminiferal survival rate, growth/calcification (mainly through test weight and SNW) and morphometric features (e.g. feeding functional structures, septal bridges, sutures and test surfaces) of the benthic foraminifer *E*. *williamsoni*.

### Live and dead foraminiferal abundance

A large number of specimens were exposed to calcein labelling, and the subsequent observation of newly deposited chambers through an epifluorescence microscope allowed easy identification of live foraminifera at the end of this initial experimental period. These methods have been widely used as research tools for foraminiferal studies in highly complex environments [45]. The use of calcein is advantageous over other labelling methods, such as the non-vital stain Rose Bengal, which may produce an overestimate of live specimens containing protoplasm.

Hence, in this study, the criterion of chamber addition (post-fluorescence growth) to distinguish live (or recently active) specimens from dead specimens of *E*. *williamsoni* is much more reliable. The results showed an extremely low percentage of surviving specimens displaying post-fluorescent growth (new chambers added) throughout the experimental period.

### Survival rate

In this study, *E*. *williamsoni* cultured at 13 °C exhibited mortality as high as 50% throughout both the calcein incubation (4 weeks) and experimental period of 6 weeks at the ambient pH of 8.1. However, mortality rate estimated for each culture conditions exhibited a considerable contribution of OA of up to 30% to total mortality at low pH/ high CO_2_ concentrations (Table 1). This highlights both the difficulty in obtaining a large number of living specimens of *E*. *williamsoni* to be analysed at the end of the experiment, and also the potential problems in maintaining a long-term foraminiferal culture, particularly under future CO_2_ scenarios.

Similar to our results, negative effects of OA on survival rates of *E*. *williamsoni* have been observed in studies in habitats with a natural pH gradient, where the abundance and diversity of benthic foraminiferal communities were significantly reduced as a consequence of low pH /high pCO_2_ and low carbonate ion concentration (CO_3_^2−^) [66,67]. Generally, under these CO_2_ scenarios, high shell dissolution rates combined with reduced calcification rates are potentially the main factors to directly influence the disappearance of calcareous species [34]. However, not all foraminiferal OA studies show negative effects on survival rate of benthic calcareous species producing low-Mg calcite tests in short-term [68]. This, in combination with our results, suggests that foraminiferal communities may show species-specific responses to future high CO_2_ concentrations.

### Foraminiferal growth and calcification

#### Biometric parameters

Biometric measurements (e.g. diameter, weight) were only taken at the end of the experiment. For that reason, measurements of maximum diameter and weight of tests (shells) of specimens across pH conditions were not used for foraminiferal growth and calcification estimations. However, both measurements were independently used for further analyses, and a significant difference in the maximum diameter was observed on specimens cultured at pH 7.7. For the remaining treatments, live specimens showed similar mean maximum diameter regardless of the pH conditions (S3 and S4 Tables).

Statistical analysis confirmed a significant difference in foraminiferal dry test weight across treatments, especially on those individuals exposed to the lowest pH levels (S3 and S4 Tables). Although not directly measured in this study, the reduction in foraminiferal weight is partly explained by the strong influence of the experimental pH conditions on the loss of test mass due to dissolution. This suggests high dissolution rates on tests of *E*. *williamsoni* specimens. Hence, relatively lighter specimens may be found as a result of both dissolution processes and the production of significantly thinner chambers walls. The latter has been described previously for individuals of *E*. *williamsoni* cultured for 8 weeks at pH of 7.6 [33]. The production of thinner test walls by live specimens cultured at the lowest pH levels may also explain the fragility of the outermost chambers, which partially collapsed during cleaning and picking processes at the end of this experiment. Further image analyses, including destructive (cross-sectional SEM) [69] and non-destructive (3D visualisation) [33,35,39], would allow quantification of the negative effects of OA on internal structures (thickness wall of recently deposited chambers).

The relationship between test weight and maximum test diameter has been previously used as an indicator of variation in growth/calcification rates of benthic foraminifera [32,34,35]. Relative changes in the slope (e.g. mainly more negative) of this relationship have been identified as a result of the direct effect of low pH levels/CO_2_ concentrations. Despite the observations of a slight difference among slopes of shell weight and diameter relationship across pH treatments in this study, these differences are not statistically significant except for treatments at pH 7.9 and pH 7.7 (S7 Fig and S5 Table).

#### Size-normalized weight (SNW) and carbonate ion concentration in seawater

A significantly positive correlation between the size-normalized weight (SNW) of *E*. *williamsoni* specimens and carbonate ion concentrations in seawater across different pH level/CO_2_ concentrations was clearly observed in this study (Fig 4). This relationship has also been described as a substantial indicator of changes of calcification rates, thickness of shell wall or density of planktonic and benthic foraminifera [4,57,58]. Here, our results suggest that the lowest mean of SNW of *E*. *williamsoni* and the lowest mean carbonate ion concentrations are consistent with the lowest shell weight observed across the lowest pH levels. This indicates a significant reduction of calcification rates during the 6 week-experimental period, and also suggests that future scenarios with lower seawater pH levels may affect significantly the foraminiferal carbonate production and carbon sink in coastal environments at mid-latitude.

#### Growth rates

Foraminiferal growth models (non-linear functions) can be estimated by a number of methods that incorporate periodical measurements of several parameters throughout an experimental period. Parameters such as the addition of new chambers [32,39,70,71], chamber volumes [39,72], biometric parameters such as diameter or weight [4,31,32,35,39,70,71,73] and size/weight relationship [32,35] have been widely used. In this study, the mean foraminiferal growth/calcification, defined as the amount the CaCO_3_ deposited in their structures, was inferred via counting of new chambers deposited at the end of the experimental period of 42 days (Fig 2). This method indicates that the mean foraminiferal growth/calcification was not significantly affected across pH treatments except at pH 7.9, where the foraminiferal chamber addition rate was greater than the remaining treatments. No significant differences in numbers of chambers added across different pH conditions were observed for *E*. *williamsoni* [33]. In contrast, previous work has shown significant differences in growth rate, via counting of new chambers added in a benthic foraminifera when exposed to similar pH conditions [32]. However, despite the similarity in pH levels, caution should be applied in any direct comparisons where the length of incubation or the species studied differ. In addition, different physical parameters such as temperature and salinity can also influence foraminiferal growth rates, again highlighting the need for caution in direct comparison between different studies. Further research is required to assess the effect of other physical parameters that may influence the optimal conditions for *E*. *williamsoni* growth.

However, existing literature indicates that in habitats with a natural gradient of calcium carbonate saturation and pH, the dominant *Elphydium spp*. shows significant differences in calcification rate as the CO_2_ concentrations increase [24]. Thus, at a pH level as low as 7.71, *Elphydium spp*. specimens are able to calcify at a much lower rate to maintain their low-magnesium calcite tests [24,74]. This was not the case for our experimental results where the growth/calcification of *E*. *williamsoni*, measured by changes in maximum diameter and the number of chambers added, was not negatively affected across the pH treatments. This fact may be explained, in part, by the presence of a small proportion of individuals with extreme values (outliers) for the measured parameters, particularly in pH treatment 7.9 (S6 Fig). These individuals may have apparently a strong influence on statistical estimates of the measured parameters for each pH condition, due to a reduced number of live specimens for analysis. Further experiments would help determine if this is consistent. However, these species are intertidal and may be acclimated to changes in environmental conditions over short-time periods (e.g. tidal), so may be able to maintain key processes, such as calcification, for the duration of the experiments.

### SEM images

The first experimental evidence of a severe effect of OA mainly on ornamentations of feeding functional structures in a long-time period (36 weeks) was demonstrated for benthic foraminifera *Haynesina germanica* cultured at a range of CO_2_ concentrations from 380 ppm to 1000 ppm [23]. Other studies, however, have also provided information on similar progressive signs of morphological alteration via dissolution and cracking processes on foraminiferal tests in short-time experimental periods under similar CO_2_ concentrations. For instance, foraminiferal calcareous species exposed to high CO_2_ concentrations exhibited a substantial stage of corrosion mainly in test surface, sutures, around the pores [31,75], and also in internal test density [35]. Similarly, SEM images of live specimens of *E*. *williamsoni* presented here indicate a progressive alteration in the foraminiferal morphology (test) when individuals were exposed to low pH/high CO_2_ concentrations for a similar short-time period. The images reveal how in the lower pH treatments the test surfaces, septal bridges, sutures and apertural regions, including feeding functional structures such as teeth and tubercles, have been compromised in comparison to those individuals cultured at pH 8.1 (ambient) and 7.9 (Fig 5). The high level of corrosion and dissolution seen in the SEM images are consistent with parameters such as SNW and mean test weight, confirming the negative effect of OA on *E*. *williamsoni*. Our results suggest that a pH level of 7.7 may indicate the threshold for this species to exhibit a significant change in biometric and morphological features with a subsequent effect on growth/calcification and survival in the short-term.

### Ecological significance

Short-term effects of OA on benthic foraminifera may be significantly important for ecosystem functional structure as these organisms play a crucial role in biogeochemical cycles in coastal environments [6]. Thus, under future high CO_2_ scenarios, a reduction in abundance and foraminiferal assemblages may contribute to the alteration of carbon cycling due to the reduction in the degradation rate of organic matter. Furthermore, the production of calcium carbonate and the ocean’s carbon sink capacity may also be affected in coastal marine habitats, although the implications of long-term OA are not yet clear.

Direct biological impacts of OA on functional feeding structures of *E*. *williamsoni* specimens may alter their common feeding/sequestration mechanisms. A similar feeding mechanism was previously described for *H*. *germanica* under ambient conditions [60]. Furthermore, these morphological alterations may lead to a reduction in foraminiferal feeding efficiency with a subsequent loss of species-specific competitiveness and ultimately affect their long-term fitness and survival [23]. Future ecological impacts of OA may suggest a future disappearance of foraminiferal species with a subsequent shift in both the foraminiferal benthic community structures and the transfer of nutrients (energy) towards multiple components of the benthic food webs. Several studies have confirmed that a shift in benthic foraminiferal composition driven mainly by OA will be highly beneficial to non-calcifying species in long-term [23,24,66]. Thus, assemblages of calcareous species naturally found at pH 8.19 may shift to communities dominated by agglutinated species at pH 7.7 [24]. Generally, the potential disappearance of one calcareous species may be directly linked to high shell dissolution rates combined with reduced calcification rates as a direct consequence of low pH levels/ high CO_2_ concentrations [31].

Despite the description of these biological impacts on benthic community structures under future high CO_2_ scenarios, the mechanisms involved in the transitional processes of ecological succession that may precede the foraminiferal disappearance of calcareous species are still unclear. Thus, within assemblages of calcareous benthic foraminifera, co-occurring species under the same unfavourable environmental conditions may show species-specific features that help one species to prevail over other calcareous species in short- and long-term. For instance, as a qualitative comparison, our results from SEM images suggest that *E*. *williamsoni* is more sensitive to high CO_2_ concentrations and low pH over short-term periods of exposure than *H*. *germanica*. The latter required a more extended period of exposure before similar altered morphology in functional structures were apparent [23]. This may indicate an ecological advantage for *H*. *germanica* over *E*. *williamsoni* due to a higher capacity to resist long-term dissolution process. Hence, under future increased CO_2_ scenarios, a greater occurrence of *H*. *germanica* over *E*. *williamsoni* may be expected in marsh ponds, drainage ditches, tidal flats and tidal channels where these two dominant species co-exist [76,77]. Furthermore, this potential shift in dominance between two co-occurring foraminiferal benthic species may be the first suggestion of the dominance of non-calcifiers in the coastal benthic sediments directly affected by ocean chemistry as a function of changes in atmospheric CO_2_.

Our results provide some insights into potential responses of one of the dominant species of mudflats habitats to future scenarios of high CO_2_ concentrations and low pH. However, we cannot determine exactly which component of the seawater carbonate system drives these observed changes, in contrast to other studies where benthic foraminifera and bivalves were clearly affected by one of the parameters of the carbonate system such as a decreased carbonate ion concentration or calcium carbonate saturation state [4,78].

It is still crucial to improve knowledge of the mechanisms by which early foraminiferal succession process is generated, as well as the time required for benthic organisms to display significant changes in their multiple biological parameters and processes. Measuring these responses on additional foraminiferal species from different environments will progress our understanding of any species-specific responses to OA conditions. Future complementary work on changes in foraminiferal feeding efficiency (uptake of nutrients) via isotopic labelling experiments is likely to significantly increase our understanding of OA effects on *E*. *williamsoni* and other co-existing species from intertidal habitats.

## Conclusion

This study provides a more detailed understanding of the impacts of OA on the ecology of a dominant benthic foraminifer and the future implications for benthic communities in intertidal mudflat habitats. Under future scenarios with high CO_2_ concentration resulting in low seawater pH; survival, growth, calcification, morphology and biometric features of *E*. *williamsoni* could be negatively affected. These negative effects may considerably affect the distribution, abundance, and biomass of *E*. *williamsoni*. This fact may imply an alteration in the energy transfer within the benthic food web and a shift in benthic community structures, ultimately affecting carbon cycling and total CaCO_3_ production, both highly significant in coastal waters.

## Supporting information

S1 Fig Sampling site, mudflats on Eden Estuary mudflats on Eden Estuary, Fife, UK. Living assemblage of *Elphidium williamsoni* observed in the recently collected sediment samples. These benthic foraminiferal specimens show their characteristic brown/yellow protoplasm extensively distributed across the entire foraminiferal tests, except in the last chambers.
(PDF)

S2 Fig Seawater recirculating system used for calcein incubation of *Elphidium williamsoni* under controlled conditions. A) A peristaltic pump (with 9 channels) is shown above the experimental mesocosms. B) A side view of flasks housing seawater with calcein and sediment containing living foraminifera. C) Specimens of *Elphidium williamsoni* showing the incorporation of calcein into the new growth of foraminiferal test.
(PDF)

S3 Fig Foraminiferal culturing system connected to the controlled recirculating seawater system. A) From the left mixing tanks with seawater bubbled with atmospheric CO_2_ concentrations of approx. 400 μatm *p*CO_2_/pH 8.1, 600 μatm *p*CO_2_/pH 7.9, 900 μatm *p*CO_2_/pH 7.7 and >2000 μatm *p*CO_2_/pH 7.3. B) Foraminiferal culturing system used for CO_2_ experiments.
(PDF)

S4 Fig Image of a live specimen of *Elphidium williamsoni* displaying newly formed chambers (darker shaded sections) deposited after the experimental period of 52 days at different pH conditions. Chambers precipitated in the last whorl were easily recognized by their characteristic non-fluorescent colour (n = 3) compared to the bright chambers that were present in the calcein incubation. White scale bar represents 100 μm.
(PDF)

S5 Fig Number of retrieved individuals (live and dead) with morphological changes observed in tests of *Elphidium williamsoni* as potential responses to experimental pH conditions. Morphological response levels were: **Level 1** = intact test (red), **Level 2** = minor changes (orange) and **Level 3** = broken test (yellow).
(PDF)

S6 Fig Distribution of individuals of *Elphidium williamsoni* in relation to A) maximum test diameter, B) dry test weight, and C) number of chambers added, for each culture condition (pH 8.1 (ambient), pH 7.9, pH 7.7 and pH 7.3). Individuals were sorted into groups of different bandwidth for each parameter. The bandwidth equals to 25 μm for size class, 4 μg for test weigh and 1 for a deposited chamber. Red vertical lines indicate the mean values.
(PDF)

S7 Fig Regression lines of the relationship between maximum log test diameter and log test weight (raw data) among pH treatments of *Elphidium williamsoni*. The different colours represent the different OA/pH treatments: black (ambient: pH 8.1/ 400 μatm CO_2_); green (pH 7.9/ 600 μatm CO_2_); red (pH 7.7/ 900 μatm CO_2_); and blue (pH 7.3/ > 2000 μatm CO_2_).
(PDF)

S1 Table Statistics of a nested one-way ANOVA conducted to test pseudo-replicates tanks effects of pH treatments on all variables tested for *Elphidium williamsoni*. Significant differences are in bold (p < 0.05).
(PDF)

S2 Table Shapiro-Wilk’s normality and Levene's homogeneity of variance tests of raw data for all variables tested for *Elphidium williamsoni*. (PDF)

S3 Table Kruskal-Wallis rank sum test of all variables tested for *Elphidium williamsoni*. (PDF)

S4 Table Pairwise comparisons using Dunn's-test for multiple comparisons of independent samples for *Elphidium williamsoni*. (PDF)

S5 Table Comparison between slopes of linearised functions of the relationship between test weight and maximum test diameter among pH treatments for *Elphidium williamsoni*. (PDF)

S6 Table Seawater and carbonate system parameters for 6–week experiments (means ± standard error). Calculated parameters were calculated using CO2SYS software (version 01.05).
(PDF)
