# Supplementary figures and images for: Impacts of ocean acidification on intertidal benthic foraminiferal growth and calcification
Source: PLoS One. 2019 Aug 21;14(8):e0220046. doi: 10.1371/journal.pone.0220046 (PMC6703850; doi:10.1371/journal.pone.0220046)

**S1 Fig**

**Intertidal mudflat on Eden Estuary**

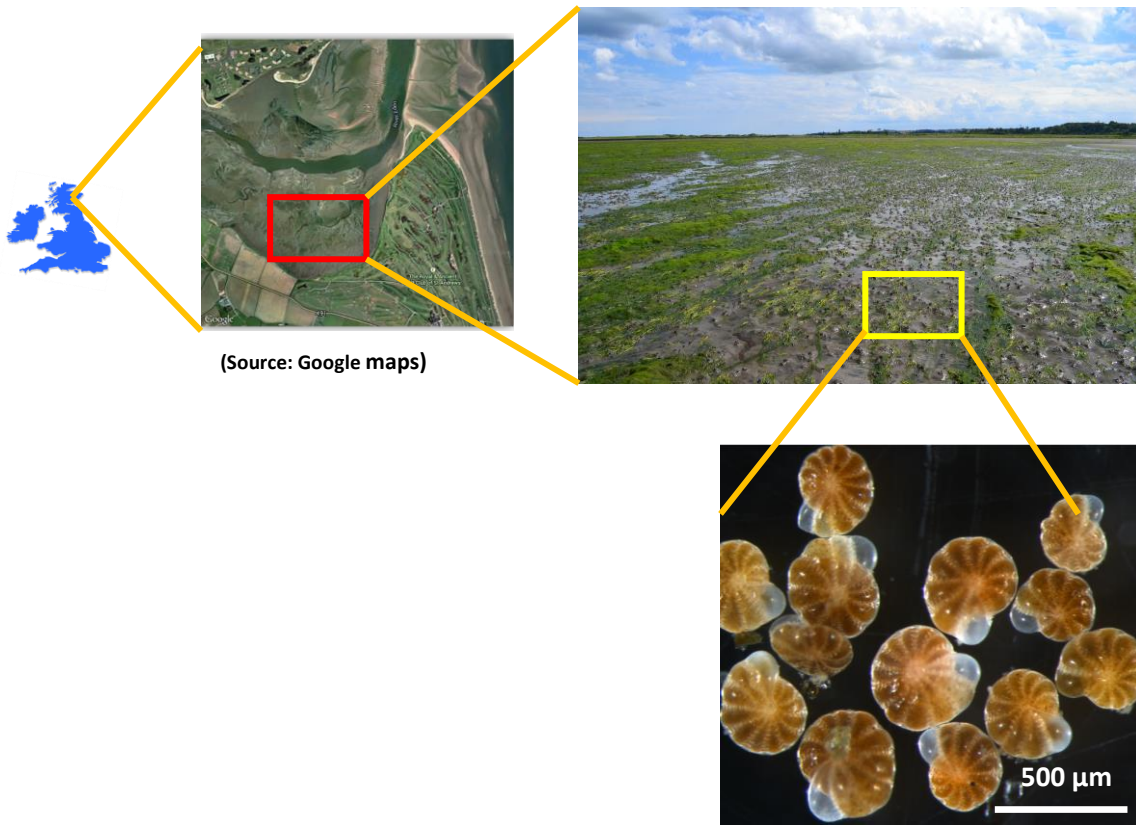

**Living assemblage of *Elphidium williamsoni***

Supplement: S1 Fig — Living assemblage of Elphidium williamsoni observed in the recently collected sediment samples. These benthic foraminiferal specimens show their characteristic brown/yellow protoplasm extensively distributed across the entire foraminiferal tests, except in the last chambers. (PDF) [file pone.0220046.s001.pdf]

S2 Fig

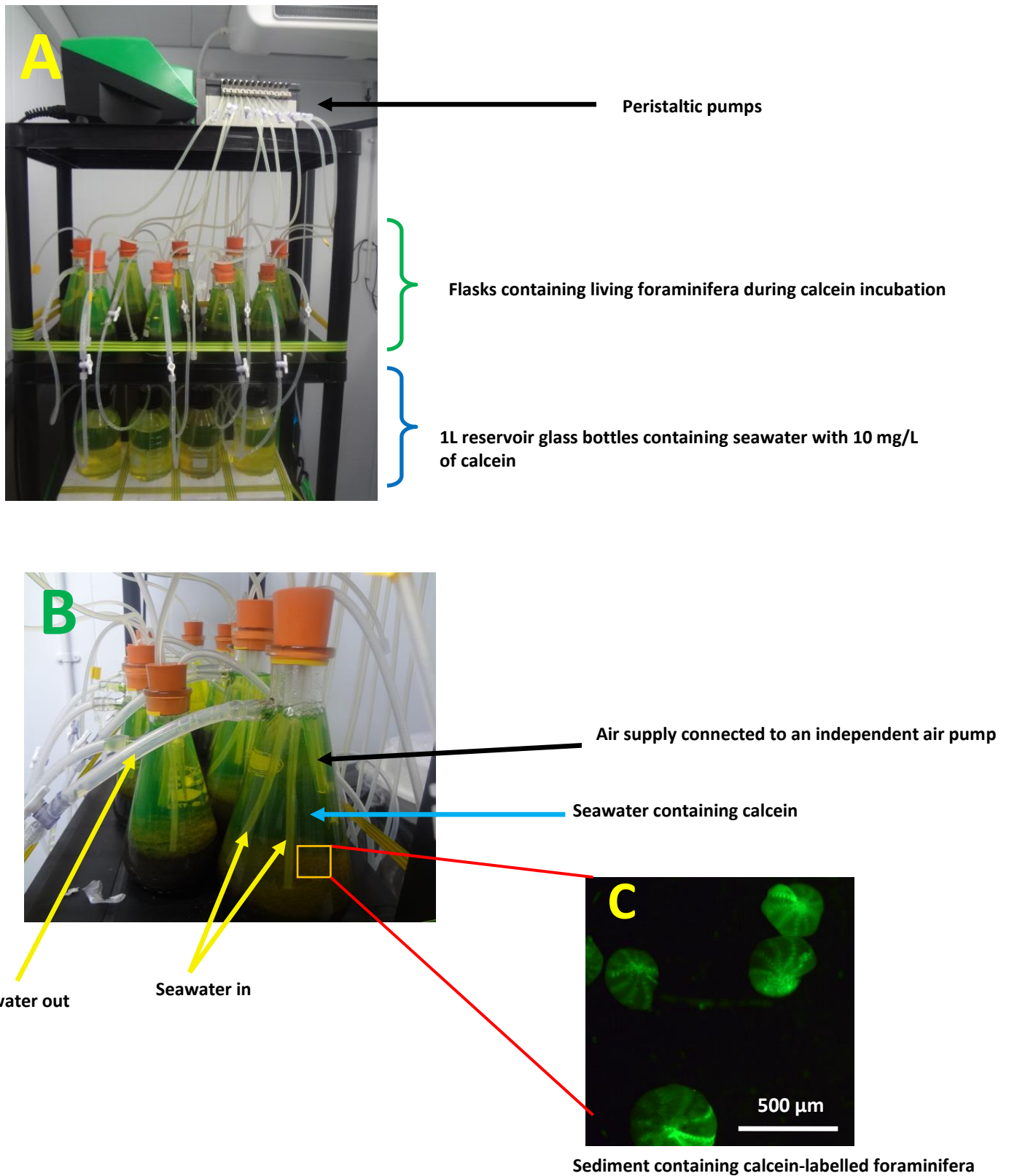

Supplement: S2 Fig — A) A peristaltic pump (with 9 channels) is shown above the experimental mesocosms. B) A side view of flasks housing seawater with calcein and sediment containing living foraminifera. C) Specimens of Elphidium williamsoni showing the incorporation of calcein into the new growth of foraminiferal test. (PDF) [file pone.0220046.s002.pdf]

S3 Fig

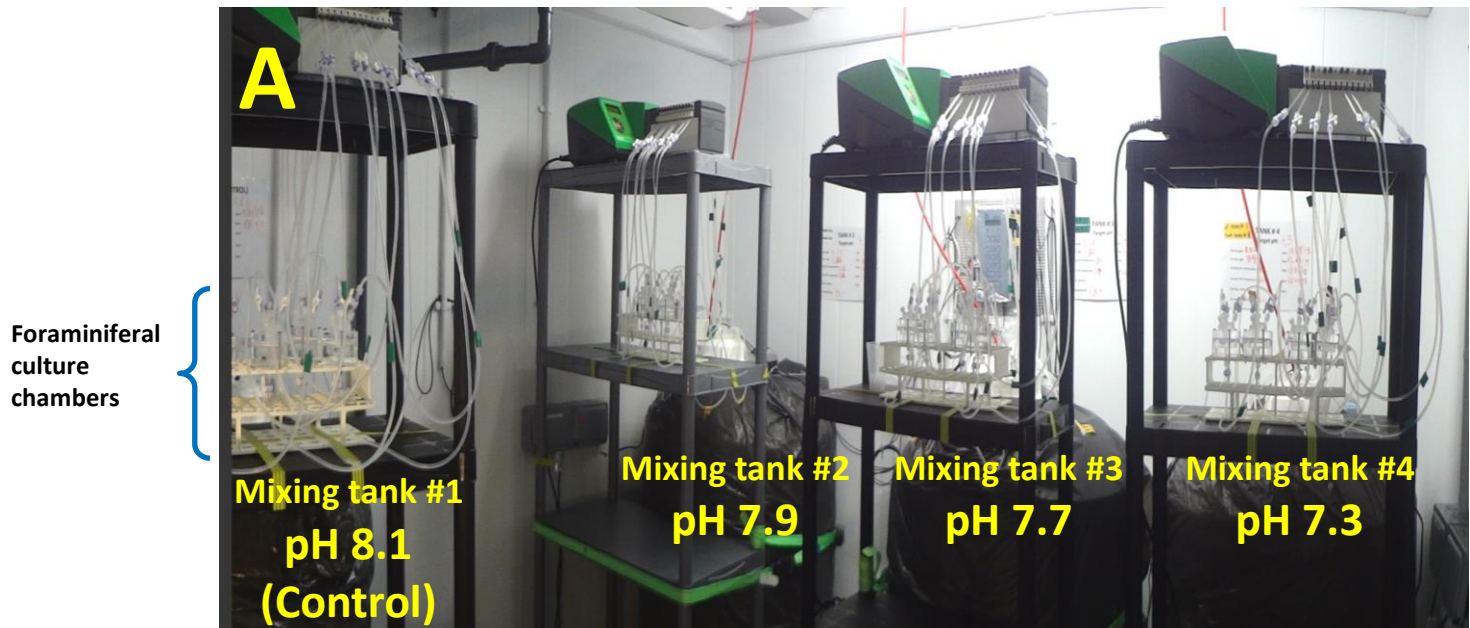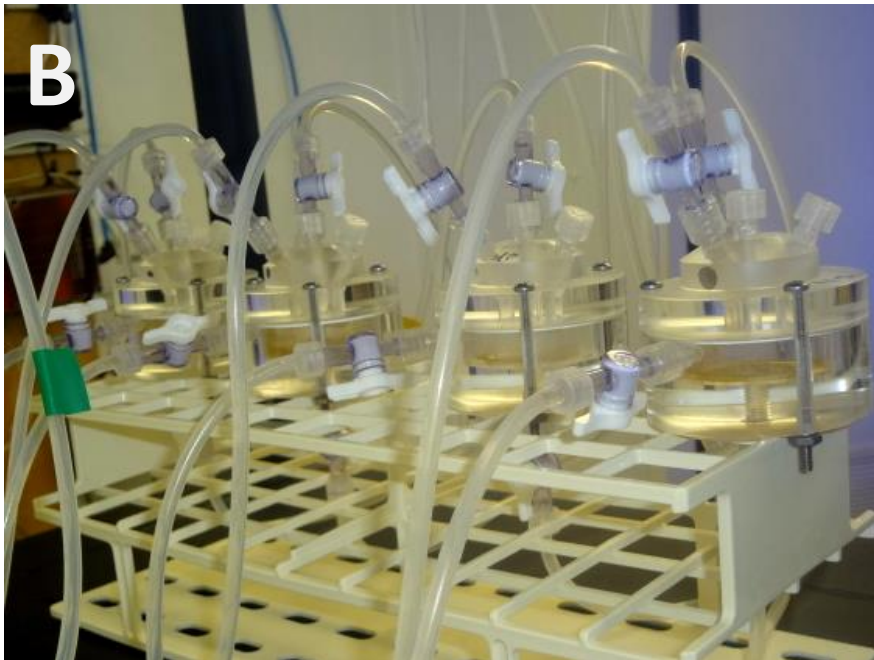

Supplement: S3 Fig — A) From the left mixing tanks with seawater bubbled with atmospheric CO2 concentrations of approx. 400 μatm pCO2/pH 8.1, 600 μatm pCO2/pH 7.9, 900 μatm pCO2/pH 7.7 and >2000 μatm pCO2/pH 7.3. B) Foraminiferal culturing system used for CO2 experiments. (PDF) [file pone.0220046.s003.pdf]

**S4 Fig**

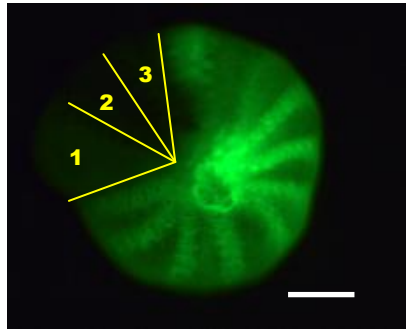

Supplement: S4 Fig — Chambers precipitated in the last whorl were easily recognized by their characteristic non-fluorescent colour (n = 3) compared to the bright chambers that were present in the calcein incubation. White scale bar represents 100 μm. (PDF) [file pone.0220046.s004.pdf]

**S5 Fig**

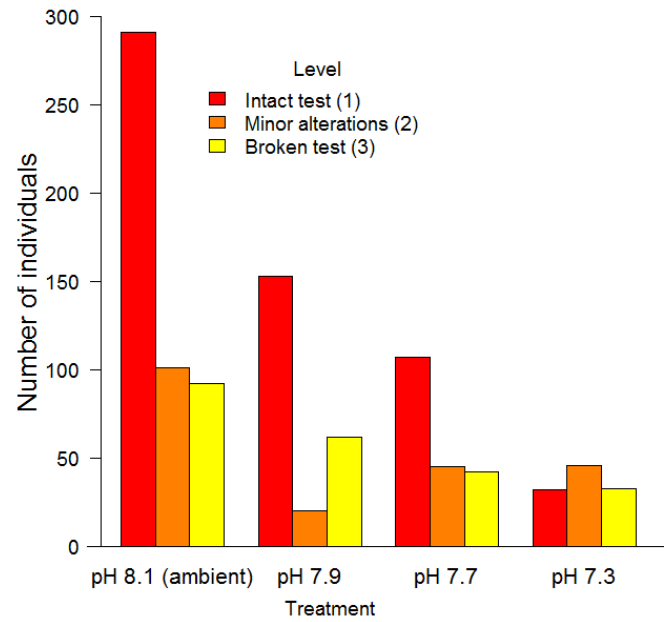

Supplement: S5 Fig — Morphological response levels were: Level 1 = intact test (red), Level 2 = minor changes (orange) and Level 3 = broken test (yellow). (PDF) [file pone.0220046.s005.pdf]

S6 Fig

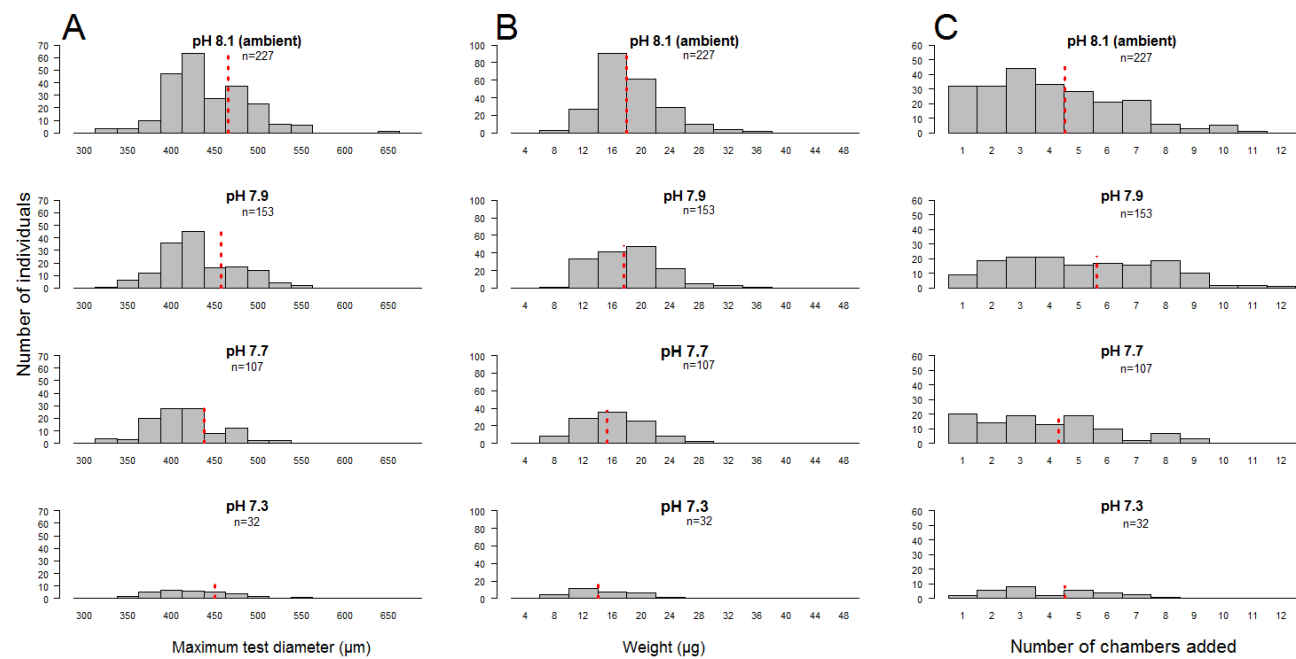

Supplement: S6 Fig — Individuals were sorted into groups of different bandwidth for each parameter. The bandwidth equals to 25 μm for size class, 4 μg for test weigh and 1 for a deposited chamber. Red vertical lines indicate the mean values. (PDF) [file pone.0220046.s006.pdf]

**S7 Fig**

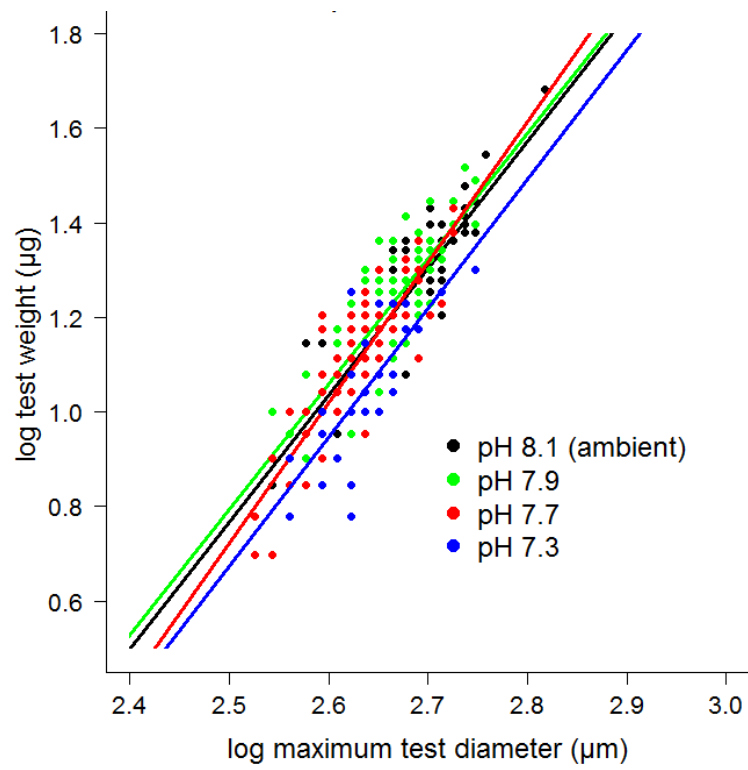

Supplement: S7 Fig — The different colours represent the different OA/pH treatments: black (ambient: pH 8.1/ 400 μatm CO2); green (pH 7.9/ 600 μatm CO2); red (pH 7.7/ 900 μatm CO2); and blue (pH 7.3/ > 2000 μatm CO2). (PDF) [file pone.0220046.s007.pdf]
