# Supplementary material for: Impacts of ocean acidification on intertidal benthic foraminiferal growth and calcification
Source: PLoS One. 2019 Aug 21;14(8):e0220046. doi: 10.1371/journal.pone.0220046 (PMC6703850; doi:10.1371/journal.pone.0220046)
Supplement: S1 Table — Significant differences are in bold (p < 0.05). (PDF) [file pone.0220046.s008.pdf]

S1 Table

Effect	DF	SS	MS	F	P
**Nested mean effect on shell diameter**					
pH	3	55973	18658	9.758	< **0.001**
Nested (pH x replicates)	12	5060	422	0.221	0.997
Residuals	503	961724	1912		
**Nested mean effect shell weight**					
pH	3	876	291.87	12.353	< **0.001**
Nested (pH x replicates)	12	292	24.36	1.031	0.418
Residuals	503	11885	23.63		
**Nested mean effect on chambers added**					
pH	3	146.5	48.83	9.482	< **0.001**
Nested (pH x replicates)	12	210.9	17.57	3.413	< **0.001**
Residuals	503	2590.1	5.15		
