# Supplementary material for: Impacts of ocean acidification on intertidal benthic foraminiferal growth and calcification
Source: PLoS One. 2019 Aug 21;14(8):e0220046. doi: 10.1371/journal.pone.0220046 (PMC6703850; doi:10.1371/journal.pone.0220046)
Supplement: S2 Table — (PDF) [file pone.0220046.s009.pdf]

S2 Table

Response variable	Test	Test value	p-value
Diameter	Normality	0.9758	1.45E-07
	Variance	0.4426	0.7227
Weight	Normality	0.9377	6.513E-14
	Variance	0.8426	0.471
Chambers added	Normality	0.9451	6.142E-13
	Variance	2.6945	0.04543
