# Supplementary material for: Impacts of ocean acidification on intertidal benthic foraminiferal growth and calcification
Source: PLoS One. 2019 Aug 21;14(8):e0220046. doi: 10.1371/journal.pone.0220046 (PMC6703850; doi:10.1371/journal.pone.0220046)
Supplement: S3 Table — (PDF) [file pone.0220046.s010.pdf]

S3 Table

Response variable	H	P-value
Diameter	28.3492	3.068E-06
Weight	35.303	1.051E-07
Chambers added	22.4055	5.37E-05
