# Supplementary material for: Impacts of ocean acidification on intertidal benthic foraminiferal growth and calcification
Source: PLoS One. 2019 Aug 21;14(8):e0220046. doi: 10.1371/journal.pone.0220046 (PMC6703850; doi:10.1371/journal.pone.0220046)
Supplement: S4 Table — (PDF) [file pone.0220046.s011.pdf]

S4 Table

Response variable	Group	meanRj	M
Diameter	pH 8.1 (ambient)	292.3943	a
	pH 7.9	257.6471	a
	pH 7.7	200.5187	b
	pH 7.3	240.3438	a,b
Weight	pH 8.1 (ambient)	283.9141	a
	pH 7.9	281.8203	a
	pH 7.7	205.5327	b
	pH 7.3	168.1562	b
Chambers added	pH 8.1 (ambient)	243.4273	a
	pH 7.9	307.1438	b
	pH 7.7	231.1495	a
	pH 7.3	248.625	a,b
