# Supplementary material for: Impacts of ocean acidification on intertidal benthic foraminiferal growth and calcification
Source: PLoS One. 2019 Aug 21;14(8):e0220046. doi: 10.1371/journal.pone.0220046 (PMC6703850; doi:10.1371/journal.pone.0220046)
Supplement: S5 Table — (PDF) [file pone.0220046.s012.pdf]

S5 Table

Comparison between slopes	Std. Error	t-value	p-value
pH 8.1 (ambient) x pH 7.9	0.0001774	0.163	0.9984
pH 8.1 (ambient) x pH 7.7	0.0002041	-1.398	0.5013
pH 8.1 (ambient) x pH 7.3	0.0003075	-0.141	0.999
pH 7.9 x pH 7.7	0.000221	1.422	0.4862
pH 7.9 x pH 7.3	0.000319	0.227	0.9959
pH 7.7 x pH 7.3	0.0003346	-0.723	0.8881
