# Supplementary material for: Impacts of ocean acidification on intertidal benthic foraminiferal growth and calcification
Source: PLoS One. 2019 Aug 21;14(8):e0220046. doi: 10.1371/journal.pone.0220046 (PMC6703850; doi:10.1371/journal.pone.0220046)
Supplement: S6 Table — Calculated parameters were calculated using CO2SYS software (version 01.05). (PDF) [file pone.0220046.s013.pdf]

S6 Table

Measured parameters					Calculated parameters					
**Treatment**	**pH (Total)**	**T (°C)**	**Salinity (ppt)**	**AT (µmol/Kg)**	**DIC (µmol/kg)**	**pCO_2 (µatm)**	**HCO_3^{1-} (µmol/kg)**	**CO_3^{2-} (µmol/kg)**	**$\Omega_{Calcite}$**	**$\Omega_{Aragonite}$**
pH 8.1 (ambient)	8.10 ± 0.02	13.17 ± 0.05	33.02 ± 0.14	2486.19 ± 118.41	2250.90 ± 114.60	380.70 ± 31.43	2063.54 ± 107.99	172.16 ± 8.60	4.15 ± 0.21	2.64 ± 0.13
pH 7.9	7.93 ± 0.04	13.2 ± 0.11	33.10 ± 0.08	2420.86 ± 60.71	2266.41 ± 52.50	578.35 ± 53.92	2122.49 ± 47.96	120.85 ± 10.97	2.91 ± 0.27	1.85 ± 0.17
pH 7.7	7.76 ± 0.03	13.2 ± 0.08	33.20 ± 0.13	2368.49 ± 54.21	2282.17 ± 55.31	885.01 ± 72.32	2164.84 ± 53.15	82.06 ± 4.77	1.97 ± 0.12	1.26 ± 0.07
pH 7.3	7.34 ± 0.02	13.2 ± 0.08	33.19 ± 0.20	2409.40 ± 103.49	2458.78 ± 106.99	2484.36 ± 162.43	2326.09 ± 100.84	33.67 ± 2.00	0.81 ± 0.05	0.51 ± 0.03
